# Cervical spine trauma

**DOI:** 10.4103/0019-5413.36985

**Published:** 2007

**Authors:** Joel A Torretti, Dilip K Sengupta

**Affiliations:** University Orthopedics Center 101 Regent Ct State College, PA 16801, USA; * Department of Orthopaedic Surgery, Dartmouth-Hitchcock Medical Center, One Medical Center Drive, Lebanon

**Keywords:** Cervical spine injuries, subaxial cervical spine trauma, upper cervical spine trauma

## Abstract

Cervical spine trauma is a common problem with a wide range of severity from minor ligamentous injury to frank osteo-ligamentous instability with spinal cord injury. The emergent evaluation of patients at risk relies on standardized clinical and radiographic protocols to identify injuries; elucidate associated pathology; classify injuries; and predict instability, treatment and outcomes. The unique anatomy of each region of the cervical spine demands a review of each segment individually. This article examines both upper cervical spine injuries, as well as subaxial spine trauma. The purpose of this article is to provide a review of the broad topic of cervical spine trauma with reference to the classic literature, as well as to summarize all recently available literature on each topic.

**Identification of References for Inclusion:**

A Pubmed and Ovid search was performed for each topic in the review to identify recently published articles relevant to the review. In addition prior reviews and classic references were evaluated individually for inclusion of classic papers, classifications and previously unidentified references.

## EPIDEMIOLOGY

It has been reported that the cervical spine is injured in 2.4% of blunt trauma victims.^1^ Certain demographic factors are known to be associated with blunt cervical spine injury: age greater than 65 years, male sex and white ethnicity.^2^ To date, only one population-based study of spinal column injuries has been performed in a complete population. Hu *et al.* reported on patients in the Manitoba Health Insurance Plan from 1981–1984.^3^ The annual incidence rate was 64/100,000 with two peaks, one in the second and third decade of the male population and another in elderly females. The most common mechanism of injury was noted to be accidental falls, with motor vehicle/transport injuries being the second most common. In another study, which is the largest multi-center trial to date, the most common site of injury was the atlantoaxial region, with the most commonly injured levels in the subaxial cervical spine being C6 and C7.^4^ One-third of the injuries identified in this study were considered clinically insignificant. Despite this surprising number of clinically minor injuries, the cervical spine remains the most common level for spinal cord injury (SCI), representing 55% of all SCIs.^5^

### Clinical evaluation/missed injuries

The reported frequency of missed injuries in the cervical spine varies from 4% to 30%.^6^,^7^ The most common reason cited for missed injuries is an inadequate radiographic examination.^8^ Characteristic injury patterns which are commonly missed include odontoid, teardrop, facet and hangman's fractures.^9^ Despite these common patterns, it has been recognized that even in the absence of fractures, clinically significant instability can exist. Spinal cord injury without radiographic abnormality has been found to occur in 0.08% of adults with blunt cervical spine trauma.^10^ When injuries are missed on initial assessment, a delay in diagnosis occurs that puts the patient at risk for progressive instability and neurologic deterioration. In one series by Davis *et al.,* 29% of patients with missed injuries developed permanent neurologic sequelae.^11^ It is clear that a systematic approach to the evaluation of suspected cervical spine injuries is important to avoid these pitfalls.

Current protocols for evaluation of suspected cervical spine injury combine information from the history, clinical examination and radiographic evaluation to predict the presence of instability, identify neurological deficits and guide the need for intervention. During the course of evaluation, patients should be maintained in a supine position with rigid collar immobilization or other stable neutral immobilization, while standard Advanced Trauma Life Support protocols are performed. The immediate clinical examination of the spine should include inspection and palpation of the spine, as well as a complete neurological examination. In addition, a cranial nerve examination should always be performed. Cranial nerve (CN) palsies related to CNs VI, VII, IX, X, XI and XII can occur in association with upper cervical spine injuries.^12^,^13^

Clinical examination of patients, although critical to all initial evaluation protocols, has severe limitations, with a sensitivity of 77% in blunt trauma patients in one series.^14^ To adequately assess a patient, he/ she must be awake and alert, nonintoxicated and without distracting injury. Patients specifically at risk for cervical spine injury include those with facial fractures/ trauma, closed head injury and blunt/ penetrating neck injury. The severity/ violence of the injury mechanism, as well as its Injury Severity Score (ISS), are important to consider and are more predictive of a significant cervical injury than other frequently described factors.^15^ Clinical protocols for determining the need for radiography have been developed, such as the NEXUS Low Risk Criteria and the Canadian C-spine Rule, which are used to aid in emergency room triage. A recent large prospective cohort study demonstrated superiority of the Canadian C-spine Rule over the NEXUS criteria with regard to sensitivity/ specificity and reducing the incidence of unwarranted radiography.^16^

### Radiographic evaluation

Once the initial trauma evaluation has been performed, it is imperative that an appropriate radiographic evaluation be obtained. The type of radiographic assessment has evolved as imaging techniques have advanced, with resultant changes in what is considered the current standard of care in many institutions. In the past, plain radiographs which included lateral, AP and odontoid views were considered the standard of care for initial trauma evaluation. Although relatively inexpensive and easy to obtain, they provided poor visualization of the craniocervical and cervicothoracic junction and resulted in missed injury rates of 15-30% in some studies.^17^,^18^ Spiral CT is widely available and has largely supplanted the role of traditional plain radiography in many institutions. The ability of spiral CT to detect subtle injuries has been demonstrated to be superior to plain radiography.^19^ Spiral CT has been found to have a sensitivity of 99% and specificity of 100%, with the risk of missed spine injury being 0.04%;^20^ while plain film radiography has a sensitivity of 70%.^21^ Spiral CT has also been shown to decrease the time in the radiology department. The cost-effectiveness of utilizing spiral CT has also been evaluated, and it has been shown to be at least equivalent, if not superior, to plain film radiography.^21^–^23^ The advantages of cervical spine spiral CT are especially apparent in the evaluation of regions poorly visualized by plain films, such as the occipitocervical region, the facets/ lamina and the cervicothoracic junction. For these reasons, it is the authors' opinion that spiral CT is the preferred method for evaluation of suspected cervical spine injuries in the acute setting.

When evaluating radiographs of patients with suspected subaxial cervical trauma, key radiographic features need to be assessed: the presence of soft tissue swelling anterior to the vertebral bodies; a loss of the normal smooth cervical lordosis with special attention to the normal lordotic lines; disc space narrowing; segmental kyphosis; antero/retrolisthesis of one vertebral body relative to another and/or splaying of the spinous processes. The information from these evaluations provides indirect assessments of spinal stability. Stability of the spine has been defined by White and Panjabi as “the ability of the spine under physiologic loads to maintain an association between vertebral segments in such a way that there is neither damage nor subsequent irritation of the spinal cord or nerve roots and, in addition, there is no development of incapacitating deformity or pain due to structural changes.” Given this framework, they have provided a scoring system that has been widely adopted in predicting the presence of instability on cervical radiographs with evidence of segmental kyphosis greater than 11 degrees and anterolisthesis greater than 3.5 mm of one vertebral body on another as strong indicators of instability.^24^ In addition to considerations of stability, a suspicion of noncontiguous spinal injury must be maintained in all patients with known cervical injuries, given a reported incidence of 15-20% of noncontiguous spinal injury,^25^,^26^ with evaluation of the remaining spine being performed according to individual institutional protocol.

### Classification

Classification systems have been developed in an attempt to predict instability, standardize the discussion of injury types and provide a means for applying a consistent approach to these injuries. These systems are divided between the upper cervical spine and subaxial cervical spine. Due to its unique anatomy the upper cervical spine has its own classifications unique to each injury pattern and level. The subaxial cervical spine has a variety of systems which each attempt to provide a comprehensive approach. One of the first and the most commonly employed systems for classifying injuries to the subaxial cervical spine is the Allen and Ferguson classification, which provides a mechanistic classification.^27^ It was proposed based upon a retrospective review of a single author's case series from 1960 to 1974. It included a description of a “major injury vector,” the inferred applied force based upon the interpretation of static radiographs, as well as a descriptive phylogeny within each vector to clarify the spectrum of injury. This resulted in the creation of six injury types: compression-flexion, compression-extension, vertical-compression, distraction-flexion, distraction-extension and lateral flexion. Each of these has a subclassification of injury types based upon their characteristic patterns.

A similar mechanistic approach was proposed by the application of the AO thoracolumbar fracture classification^28^ to the subaxial cervical spine. This was also based upon the review of an author's case series; and despite a few basic differences in some of the fracture patterns and predictions of instability, it was found to provide a reasonable framework for classification of these diverse injuries.^29^ This system groups the injury patterns into three major groups: type A (14.7%), which are “compression only” lesions of the vertebral body; type B (43.9%), which are flexion-extension-distraction injuries (representing anterior and posterior element injury with distraction); and type C (41.2%), which are rotational injuries. Despite type-A injury pattern being the predominant pattern in thoracolumbar injuries, it is the least common pattern in the subaxial cervical spine. The most common patterns are types B and C.

More recently there is a novel system proposed by Moore *et al.,*^30^ which adopts a morphologic, rather than a mechanistic, approach to the classification of the injury. They divide the spine into four columns: the anterior column; the lateral columns, consisting of the lateral masses and paired facets; and the posterior column, the posterior bony arch and supporting ligaments. Each column is then scored based upon the severity of injury. Their scoring system was found to be reproducible in their initial case series, although the utility of this approach will need to be evaluated in further studies.

Currently, the Allen and Ferguson classification is the most commonly employed method and the most practical in clinical applications for the cervical spine, where it provides a framework for interpretation of the injury mechanism and anticipated instability. Most surgeons consider injuries within the context of these classification heuristics but do not depend on their specific subclassification to predict the treatment. Rather, they use a combination of the predicted stability of an injury, the presence of neurological deficit and the injury pattern to guide their management decisions.

### Functional anatomy

**Figure 1:**
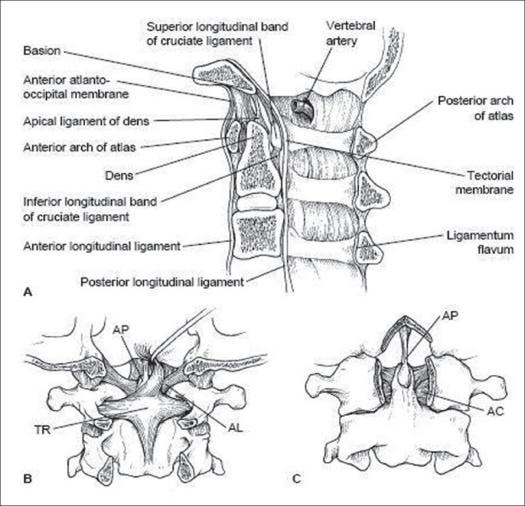
Upper cervical spine anatomy. A. Sagittal view; B. Posterior view; C. Anterior view (with anterior arch of C1 cut away)^34^

The upper cervical spine has unique anatomic features that distinguish it from the remainder of the cervical spine [Figure 1]. Its motion segments make up a large amount of total cervical spine motion and, as a result, predispose it to a unique set of injuries. The occipitoatlantal range of motion is 25 degrees in flexion-extension and 5 degrees in rotation, whereas the atlantoaxial range of motion makes up a large proportion of the cervical motion in rotation, accounting for approximately 40-50% of cervical rotation. The atlantoaxial articulation also contributes 20 degrees to the flexion-extension range of motion.^31^ The occipital condyles project laterally from the base of the skull and articulate with the facets of the atlas (C1), forming paired synovial joints. The atlas has rather shallow facets, resulting in less constraint than other facet complexes; but their capsules are reinforced by the paired alar ligaments, which are unique to this level. The alar ligaments extend cephalad from the dens to the medial aspect of the occipital condyles as paired structures. They limit the axial rotation, as well as the side bending of the occipitoatlantoaxial complex.^32^ The cephalad extension of the anterior longitudinal ligament (ALL) and the posterior longitudinal ligament (PLL) also provides ligamentous constraint to the occipitocervical complex with their contiguous extensions, which are called the anterior atlanto-occipital membrane and tectorial membrane respectively. The atlas is also constrained by the attachments of the ALL and longus colli muscles anteriorly and the ligamentum nuchae posteriorly. The vertebral arteries are paired structures which exit the foramen transversarium cephalad to C1 and travel along the rostral aspect of its posterior arch in a groove before traveling medially and cephalad into the foramen magnum. The atlas articulates anteriorly with the dens via a synovial articulation which is reinforced by the transverse ligament. This ligament is the primary constraint against anterior translation of C1 on C2. The atlas also articulates with the axis via paired synovial facet joints, which have capsules that contribute to their stability.^33^ It is the unique anatomy of these vertebrae that allows for their coupled motion in rotation, flexion-extension and lateral bending, whilst protecting the spinal cord, paired vertebral arteries and cranial nerves as they traverse this region.

The subaxial cervical spine has consistent anatomic features between its levels until the cervicothoracic junction, where there is a transition from a relatively mobile segment to a rigid one. The anterior column provides support and stability, which is a function of the vertebral bodies, intervertebral discs and the attachments of the ALL and PLL. The vertebral body carries two-thirds of the vertebral load.^34^ The posterior bony elements include the lamina, facets and spinous processes. They provide attachments for the capsuloligamentous structures, which include the supraspinous and interspinous ligaments, ligamentum flavum and facet capsules. These structures contribute to stability by providing resistance to tensile forces and are commonly described as creating the posterior tension band. The facet joints provide the primary restraint against anterior subluxation. The range of motion in the lower cervical spine is greatest at C4-5 and C5-6, although the relative contribution of each level is fairly evenly distributed amongst all the levels. The transverse foramen is located in the transverse processes and provides the conduit for the vertebral arteries bilaterally, beginning at C6.

The anatomic features of each cervical motion segment predispose the levels to different patterns of injury and, as a result, require an evaluation of each individually.

### Upper Cervical Spine Injuries (Base of skull-C2)

#### Occipitocervical dissociation

**Figure 2:**
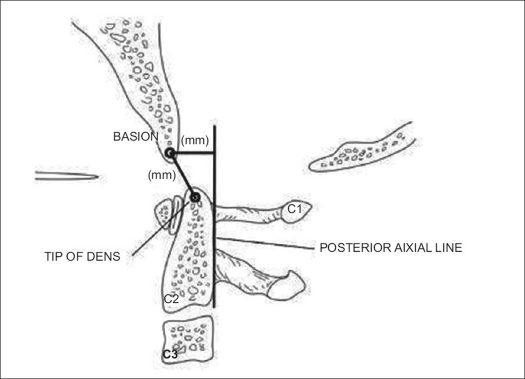
Line diagramme shows Harris Rule of 12's. Illustration for calculating the basion-dens interval (BDI) and the basion-axis interval (BAI)^57^

Occipitocervical dissociation is an uncommon injury which can be difficult to identify and has a high mortality. The most common mechanism of injury is that of a pedestrian struck by a car, with a high incidence in pediatric patients. Various radiographic parameters have been described for determining the presence of occipitoatlantal subluxation/ dislocation, with the most wellknown method being the Power's ratio (BC/ OA).^35^ This is calculated as the ratio of the distance from the basion (B) to the posterior arch (C) of C1 to the distance from the anterior arch of C1 (A) to the opisthion (O). A ratio of BC/ OA greater than 1.0 indicates anterior subluxation. A more reliable method for assessing the presence of craniovertebral dislocation is the Harris rule of 12's.^36^,^37^ Harris described a line drawn cephalad from the posterior body of C2 (posterior axial line). The distance from the basion to the posterior axial line (basion-axis interval) and the distance from the basion to the tip of the dens (basion-dens interval) should each be less than 12 mm [Figure 2]. An increase in this distance indicates instability. The classification of these injuries is based upon the displacement of the occiput. Type I injuries are anterior subluxations and are the most common. Type II injuries have vertical distraction greater than 2 mm of the atlanto-occipital joint. Type III injuries are posterior dislocations and are rarely reported.^36^ Once an injury is identified, prompt management is of the utmost importance. Traction is contraindicated. Treatment consists of immediate halo vest application with reduction of the subluxation and confirmation by CT scanning. An occiput-to-C2 fusion is required in most cases to provide longterm stability. This can be accomplished by use of a wiring technique, such as the Bohlman Wire technique, although this requires the use of a halo postoperatively. Another option is to perform rigid fixation with a plate-screw construct or a screw-rod construct. Various systems are now available that allow rod attachment to occipital plates, with case reports of their utility.^38^ The advantage of these methods is that immobilization in a rigid cervical collar is all that is required. A method of fixation from the occiput to the lateral mass of the atlas has been described but awaits further evaluation.^39^

#### Occipital condyle fractures

Occipital condyle fractures have previously been viewed as relatively uncommon injuries; but with the increased utilization of CT scanning with reconstructions in the evaluation of suspected spine trauma patients, an increased incidence has been noted. It has been reported to occur in 3-15% of trauma patients.^40^–^42^ The most commonly employed classification system for these injuries is that proposed by Anderson and Montesano.^43^ They described three types. Type I is an impaction fracture, which is a result of axial loading and lateral bending. This injury is not considered to be unstable. Type II is a basilar skull fracture that extends into the occipital condyle. This is also a stable injury, given that the alar ligaments and the tectorial membrane are intact. A type III occipital condyle fracture is a tension injury, resulting in an avulsion of the occipital condyle. If there is associated disruption of the alar ligaments and tectorial membrane, then the potential for instability exists. For this reason, a type III fracture is considered potentially unstable. Type I and II fractures are typically treated conservatively with immobilization in a rigid cervical collar for 6–8 weeks. Type III fractures should be treated with halo-vest immobilization if there is a suspicion of ligamentous instability, although this can be difficult to determine accurately in some injuries. If there is evidence of craniovertebral subluxation, some authors advocate immediate occiput-to-C2 fusion.

### Atlas fractures

**Figure 3:**
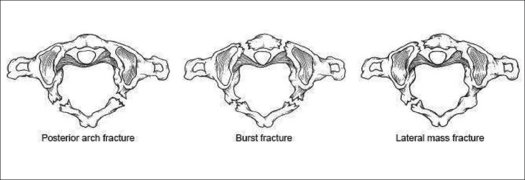
Line diagramme shows common atlas fracture patterns^47^

**Figure 4:**
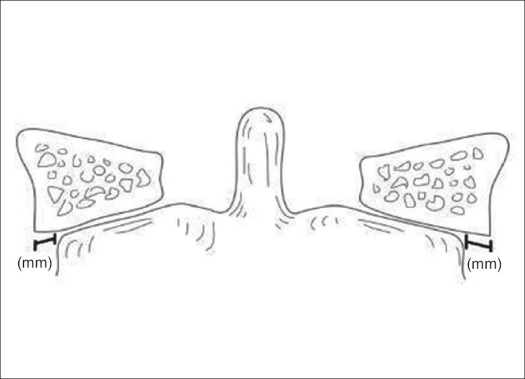
Line diagramme shows method for calculation of lateral mass overhang^57^

Atlas fractures are common cervical spine fractures, constituting 10% of all cervical spine fractures.^44^ They have a high incidence of association with other cervical spine fractures. These fractures are classified based upon fracture location [Figure 3]. The ring of C1 is commonly described as having three constituent parts: the anterior arch, the posterior arch and the lateral masses. Posterior arch fractures are typically bilateral, are the most common and are stable. Lateral mass fractures are usually unilateral and may have instability if there is associated ligamentous injury. The burst fracture is commonly called a Jefferson fracture and has a characteristic pattern of fractures in both the anterior and posterior arches. In evaluation of this type, it is imperative to assess for excess displacement of the lateral masses on an open-mouth odontoid radiograph or coronal CT reconstruction. If the sum of the lateral mass overhang is greater than 6.9 mm, then disruption of the transverse ligament can be assumed^45^ [Figure 4]. In nondisplaced or minimally displaced fractures, a cervical orthosis for 8-10 weeks is all that is required.^46^ In burst fractures with instability or significant displacement, different treatments have been utilized. Traditional treatment is by bed rest with traction for 4-6 weeks to reduce the lateral mass displacement, followed by halo vest application for mobilization.^47^ Alternatives include reduction with traction, followed by C1-2 transarticular screw fixation using the Magerl technique^48^,^49^; this method precludes the need for prolonged recumbency and is the preferred method in many institutions. Another method, which has been described in a small case series consisting of six patients, is an open transoral reduction with osteosynthesis of C1 anterior ring and lateral masses.^50^ This technique theoretically spares the atlantoaxial joints in young patients, although the widespread applicability has yet to be substantiated.

#### Atlantoaxial rotatory instability

Atlantoaxial rotatory instability is an uncommon injury in adult patient population. It is typically a result of traumatic injuries and is often associated with other upper cervical spine fractures.^51^,^52^ Most of the descriptions and methods for evaluation are a result of case reports in a nontraumatic pediatric population; and as a result, the applicability is limited in the adult trauma patient. The normal constraints to excessive atlantoaxial instability are provided by the alar and transverse ligaments. These injuries often are missed in initial evaluation and present late with pain, torticollis and limited head rotation. Fielding and Hawkins proposed a classification of this infrequently diagnosed entity, which divided it into four types:^53^ Type I – rotatory fixation without anterior displacement of the atlas; Type II – rotatory fixation with anterior displacement of the atlas of 3-5 mm; Type III – rotatory fixation with anterior displacement greater than 5 mm; and Type IV – rotatory fixation with posterior displacement. In Type I injuries, there is no ligamentous disruption; but in the other types there is, by definition, rupture of one or more of the ligaments. The classification is limited in that it does not provide for the rare entity, which has been described, of associated atlanto-occipital subluxation/fixation.^54^ When an injury of this sort is suspected, CT radiography is the primary imaging modality; but diagnosis can still remain elusive, given the difficulty in interpreting the images in a patient whose study was acquired in a rotated/ tilted position. Dynamic CT scanning may increase the diagnostic yield in patients with a Type I lesion,^55^,^56^ although this is not typically recommended in traumatic injuries. Treatment is aimed at reduction with traction. If it is stable following reduction, then halo application is considered the standard of care. If the injury proves to be unstable or is a late presentation, the options are an open reduction and posterior stabilization versus stabilization *in situ.* This can be accomplished with a variety of posterior C1-2 fusion methods, including a Gallie-type fusion, Magerl transarticular screws or the Harms technique utilizing C2 pedicle screws. The method is at the discretion of the surgeon, although individual patient factors, surgical risk and an individual surgeon's experience will guide the choice.

#### Atlantodens instability

Atlanto-dens instability is a result of rupture of the transverse ligament and occasionally the alar ligaments and tectorial membrane. It is typically the result of a flexion injury. It is assessed by a measurement of the anterior atlanto-dens interval. In adult patients, up to 3 mm is considered normal. The posterior atlanto-dens interval is a useful tool for measuring the canal diameter and has demonstrated utility in rheumatoid patients but does not have any published prognostic value in the trauma population.^57^ When an injury is suspected, CT radiography allows identification of subluxation and provides a thorough evaluation for potential associated fractures. MRI scanning will allow identification of ligament rupture in many cases, as well as an evaluation of other ligamentous structures. Treatment is directed at stabilization. Halo immobilization does not provide a reliable treatment, given the poor healing potential of these injuries.^36^ For this reason, it is recommended that individuals with significant instability undergo a C1-2 fusion using one of the aforementioned methods.

### Odontoid fractures

**Figure 5:**
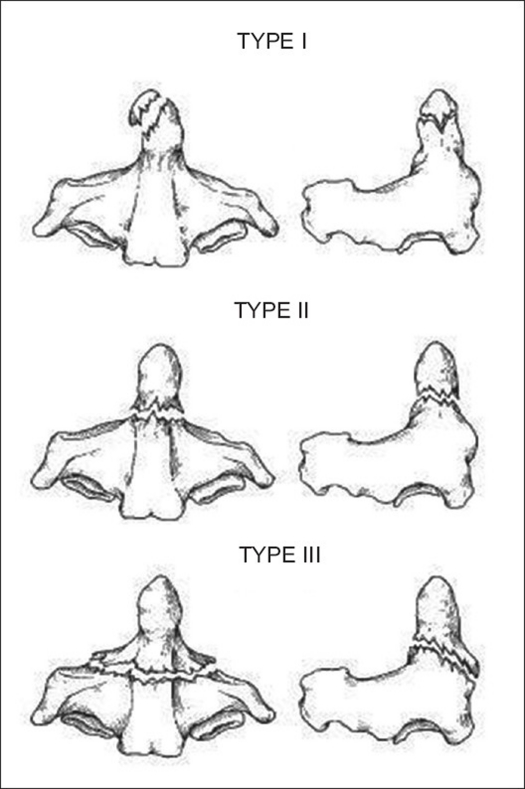
Line diagramme shows odontoid fracture classification^58^

Odontoid fractures are common cervical spine fractures, representing up to 20% of all cervical spine fractures in some studies.^58^,^59^ They have a bimodal incidence, with the first peak occurring in young patients in association with high-energy trauma; and the second peak occurring in elderly patients in association with low-energy mechanisms, such as falls. These injuries commonly have no neurological involvement, although a spectrum of injury – from mild upper extremity weakness to complete quadriparesis – can be seen. The classification of these injuries was proposed by Anderson and D'Alonzo^58^ and is based upon the location of the fracture line [Figure 5]. Type I fracture is the least common fracture pattern and occurs at the tip of the odontoid. Type I fractures are thought to be stable injuries, although an evaluation for associated instability is imperative as these can be seen in association with occipitocervical instability as an alar ligament avulsion. Type II fractures are the most common. The fracture line is at the junction of the odontoid base and the body. Type III fractures are fractures of the odontoid which extend into the body of C2. Prognosis and rate of union are closely related to both the fracture type and degree of displacement. Type I fractures have a high union rate and may be treated conservatively in majority of the patients, granted that there is no associated instability. Type II fractures have the highest incidence of nonunion, with rates of 12-63% being reported in different series.^60^,^61^ Type III fractures have a much higher union rate, with only 8% going on to nonunion. Factors associated with higher rates of nonunion are age >65 years, smoking and displacement greater than 5 mm or angulation greater than 10 degrees. For this reason, a more aggressive initial treatment has been advocated in selected patients. Halo immobilization has been considered the standard of care, although its applicability to both trauma patients with associated head and/ or chest injuries and the elderly population is limited.^62^,^63^ An alternative is anterior odontoid screw fixation following reduction with traction. The outcome of this procedure has been shown to be successful with union rates of 75-100% and no significant difference between single- and double-screw fixation.^64^–^67^ The advantages of this approach are that patients can be mobilized in a collar, there is no risk of re-displacement, it preserves atlantoaxial motion and it is a commonly utilized surgical approach. The disadvantages are that it cannot be utilized unless the fracture pattern has the appropriate orientation, if there is significant comminution of the fracture, if there is lytic osteopenia of the dens or if precluded by a patient's body habitus and cervical kyphosis. In the appropriate patient, odontoid screw fixation is the method of choice for treatment of these common fractures and has supplanted halo immobilization. A final option that has been advocated is a posterior atlantoaxial fusion using a Gallie-type wiring method, Magerl transarticular screw fixation or a Harms fusion. All of these have been reported to have high union rates, although there are biomechanical advantages to the latter two approaches.^68^–^69^ The disadvantages include the morbidity of a posterior surgical procedure in an elderly patient and loss of rotation. Given the myriad of potential surgical approaches, the surgeon's familiarity and comfort with each will determine the optimum treatment in each case; although their use in the authors' practices is reserved for dens fracture nonunions in symptomatic patients.

### Traumatic spondylolisthesis of the axis

**Figure 6:**
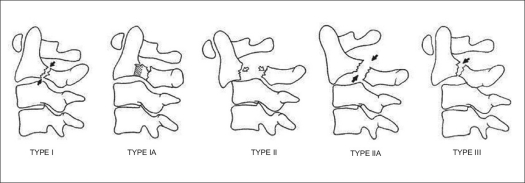
Line diagramme shows classification of traumatic spondylolisthesis of the axis^71^

Hangman's fracture is a term frequently used to describe traumatic spondylolisthesis of the axis; although the appropriateness of this term, which hearkens to the era of judicial hangings, has been questioned. This fracture is typically a result of high-energy trauma, and its most common mechanism is hyperextension and axial loading. It is commonly seen in association with motor vehicle accidents. They are rarely associated with neurological deficits. The original classification of this injury was proposed by Effendi^70^ and later modified by Levine^71^ [Figure 6]. This classification has four primary types with one subsequent addition – the “atypical” pattern. Type I is a bilateral pars fracture with a vertical fracture line, less than 3 mm of displacement and no angulation. Type II injuries have a vertical fracture line with displacement of greater than 3 mm and significant angulation. There is often an associated fracture of the anterior and superior endplate of C3. Type IIa fractures differ from Type II fractures in that they demonstrate an oblique fracture pattern of the pars, with no displacement but significant angulation – typically greater than 15 degrees. The importance of this pattern is the proposed injury vector. It is thought to be due to a flexion-distraction moment with resultant disc disruption and rupture of the PLL. As a result, traction is contraindicated for reduction. This is the least common pattern, representing 10% of “hangman's” fractures. A Type III fracture is a Type I fracture with bilateral C2-3 facet dislocations. A final type has been described more recently by Starr and Eismont and is considered an “atypical” fracture pattern, in which the fracture propagates through the posterior body of C2, rather than the pars.^72^ This has been labeled Type Ia. Treatment of Type I and Ia fractures is typically only collar immobilization. Type II and IIa fractures require reduction before immobilization. In Type II fractures, this is achieved with traction followed by halo application. It has traditionally been advocated that reduction of displaced Type II fractures be followed by 4-6 weeks of bed rest and traction prior to mobilization. This has recently been evaluated, and it was demonstrated that patients with Type II fractures with angulation of less than 12 degrees could be successfully mobilized acutely in a halo.^73^ Another alternative to prolonged recumbency is immediate operative stabilization once a reduction is achieved. This can be performed with a variety of methods. One method is direct osteosynthesis of the fracture with transpedicular lag screws. The disadvantage to this approach is that it does not address any potential instability of the disc space. An alternative is to perform an anterior C2-3 arthrodesis, but this leaves the posterior fracture unaddressed. A final method is to perform a posterior lag screw fixation of C2 with C3 lateral mass screws. A biomechanical comparison of these methods was recently performed, and both C2-3 rod construct and anterior plating were found to provide significantly greater stability to the injured segment than pars screws alone.^74^ It is the authors' practice to mobilize patients acutely in a halo and surgically stabilize ones that develop recurrent displacement, with an anterior approach being the most commonly employed method. A prospective comparison of clinical outcomes has yet to be performed, but retrospective case series indicate relative clinical equivalence between the various stabilization methods for unstable fractures.^75^ Type III injuries are felt to be the only absolute surgical indication in management of traumatic spondylolisthesis of the axis. These require an open reduction and stabilization using one of the aforementioned methods. The union rate of Type I fractures is close to 100%. Type II fractures have the possibility for nonunion, depending on the degree of initial angulation/ displacement.

### Subaxial cervical spine trauma (C3-T1)

Subaxial cervical spine injuries represent a broad array of injury patterns and degrees of instability. The current classification systems that are most commonly employed are mechanistic classifications, which, while useful for categorizing the injury patterns, do not reliably predict stability and management. For this reason, the discussion of specific injuries will review the potential for instability and management approaches for each common pattern of injury.

#### Flexion injuries

##### (a) Flexion-compression injuries

Flexion-compression injuries are one of the major classification groups proposed by Ferguson and Allen and represent a continuum of injury patterns, with minor degrees of trauma producing simple vertebral body compression fractures and more severe injuries resulting in a triangular “teardrop” fracture or a quadrangular fracture with posterior ligamentous disruption.^27^ The most severe pattern results in posterior subluxation of the posterior vertebral body into the canal; acute kyphosis; and disruption of the ALL, PLL and posterior ligaments. The rate of spinal cord injury in Allen and Ferguson's^27^ compressive flexion series was noted to range from none in the mildest injury pattern to 91% in the most severe. As a result, it is difficult to generalize treatment recommendations for these broad categories. Treatment is dependent upon the need for decompression, restoration of stability and maintenance of normal alignment. In the mildest forms of injury, simple collar immobilization is adequate. An MRI is useful in more severe injury patterns to assess the intervertebral disc and ligamentous structures. Stabilization may be obtained with halo-vest immobilization or may require operative anterior, posterior or combined approaches based upon the surgeon's determination of instability and need for decompression. A recent retrospective cohort study evaluated the mean kyphosis and outcome of treatment in patients treated with halo vest versus anterior corpectomy and plating. The operative group had an improved mean kyphosis with no major operative complications.^76^ Mild injuries are treated by the authors in a collar; while more severe injuries are treated with an anterior approach, corpectomy, anterior column restoration with allograft or a cage and plating.

##### (b) Flexion-distraction injuries

Flexion-distraction injuries also represent a spectrum of pathology from mild posterior ligamentous sprains to bilateral facet dislocations. These are the most common injury patterns in Allen and Ferguson's^27^ classification. The mildest form of injury in this class is facet subluxation and can be missed on initial evaluation. As a result, it can occasionally present as late occult instability, due to the poor healing potential of posterior ligamentous injuries. Unilateral facet dislocations and facet fracture-dislocations represent the next pattern seen in the spectrum of injury. They typically present with translation of 25% of one vertebral body on another and have a pathognomonic “sail” or “bow tie” sign on lateral radiographs.^36^ C6-7 is the level most commonly affected, and it often has neurological signs of unilateral nerve root compression; although they can manifest varying degrees of spinal cord injury. Bilateral facet dislocations have a higher incidence of neurologic injury. These injuries require reduction with traction. Before undertaking closed reduction, it is imperative that the patient be awake, alert and cooperative so that neurological status can be monitored. If the patient is not able to provide an examination during reduction, some authors recommend a prereduction MRI; although this is controversial. It has been demonstrated that acute reduction can be performed safely without a risk of neurological deterioration.^77^,^78^ Closed reduction is typically recommended by starting with 10-15 pounds and gradually increasing the weight with frequent radiographs and neurological checks. It has been demonstrated that up to 140 pounds can safely be applied in obtaining a reduction.^79^ At times a closed reduction is not possible. In these circumstances, an open reduction may need to be performed. This can be accomplished with either an anterior or posterior approach. Once a dislocation is reduced, operative stabilization has been demonstrated to be superior to nonoperative management in maintaining a reduction.^80^ It has also been shown that patients with a nondisplaced facet fracture with less than 1 mm of diastasis can be managed with an orthosis and close radiographic followup. A ligamentous injury or larger facet fragment with displacement may warrant operative stabilization. A recent study by Spector *et al.* evaluated factors on CT scanning that correlated with failure of nonoperative management.^81^ They found that unilateral facet fractures that involved greater than 40% of the absolute height of the intact lateral mass or fragments that were >1 cm were at increased risk of failure of nonoperative treatment. Operative stabilization can be performed anteriorly with diskectomy and plating or posteriorly with lateral mass screws fixation or facet/ spinous process wiring. The advantages of anterior stabilization are that it allows removal of a disc herniation and may save a fusion level. Posterior stabilization restores the posterior tension band but typically requires an additional level of fixation. A biomechanical comparison in a cadaver injury model found that lateral mass plating reduced the range of motion in the injury segment fourfold relative to anterior plating,^82^ implying a much more stable construct with the posterior fixation. Results of operative stabilization have been reported to be variable. A recent radiographic evaluation of facet injuries by Johnson *et al.* demonstrated a loss of fixation in 13% of flexion-distraction injuries, including both unilateral and bilateral facet injuries, treated with anterior plating.^83^ Failure correlated to the presence of endplate compression fractures and facet fractures. As a result it is difficult to generalize a treatment algorithm for all patients with these diverse injuries; rather, specific characteristics of the individual patient and surgeon experience will dictate the most prudent approach.

#### Vertical compression injuries

Vertical compression type injuries in the subaxial cervical spine are most commonly manifested as a cervical burst fracture. They are classified as the most severe pattern in the Allen and Ferguson vertical compression phylogeny and are classified as A3 lesions in the AO classification, representing 9.7% of all subaxial cervical spine fractures.^29^ The pattern of injury with these fractures is unique to the cervical spine, due to its lordotic nature and the relatively small spinal canal. Axial loading of the cervical spine results in compression of the vertebral body with resultant retropulsion of the posterior wall into the canal. The presence of a flexion moment can contribute to a posterior ligamentous injury, and the identification of this on routine imaging can be difficult. As a result, patients can present with a wide array of clinical patterns, depending upon the amount of canal encroachment and instability. The need for operative decompression in patients with significant instability, neurologic deficits and significant neurological compression is clear, but patients with a normal neurology and unclear radiographic risk of instability present a dilemma. Koivikko *et al.* described a retrospective cohort study of patients with cervical burst fractures treated operatively and nonoperatively.^84^ They included patients both with and without neurological injury. Operatively treated patients had a better Frankel grade, diminished kyphosis and lesser spinal canal encroachment compared to nonoperatively treated patients. Based upon the results of this study, it would appear that anterior decompression and stabilization is superior to halo-vest immobilization and is the treatment of choice, although further prospective randomized studies are required to definitively answer this question.

#### Extension injuries

Hyperextension injuries of the cervical spine are commonly described in regard to a few specific injury patterns. A stage I lesion in the classification of Allen and Ferguson is manifested by abnormal widening of the disc space, representing disruption of the ALL and disc. A stage II lesion was seen when the posterior ligaments were disrupted and the cephalad vertebrae was displaced into the spinal canal. These represent approximately 8% of all subaxial cervical spine injuries.^85^ While these injury patterns are well documented, identification of the DE I (distractive extension) lesions can be difficult and can lead to late instability if missed; and both DE I and II lesions suffer from a paucity of literature to guide treatment recommendations. Currently it is recommended to approach DE I lesions with an anterior reconstruction using a plate and graft to restore the normal tension band; and to treat DE II lesions with a combined approach, using a posterior approach first, to obtain reduction. Of particular concern is the subgroup of patients with an ankylosed cervical spine who are at risk for this pattern of injury. These are often patients who have Disseminated idiopathic skeletal hyperostosis (DISH) or ankylosing spondylitis. They are at substantial risk for devastating spine injuries with relatively low energy trauma as a result of the long lever arms created as a result of their bony ankylosis. Any of these patients with neck pain after a minor trauma should undergo an MRI if no fractures are identified on standard imaging. Operative stabilization or halo-vest immobilization is the mainstay of treatment, as conservative measures are unlikely to be successful.

Another common pattern of injury in extension injuries is central cord syndrome. This was first described by Schneider *et al.*^86^ in 1954. They described an entity with greater motor impairment of the upper extremities than that of the lower extremities with concomitant bladder dysfunction and variable sensory disturbance. They proposed that it was a result of an extension injury, with resultant spinal cord injury due to compression between a hypertrophied spondylotic disc-osteophyte complex and a bulging ligamentum flavum. This pattern of injury is commonly observed in the spondylotic spine in association with low-energy mechanisms, such as a fall from standing height, although it can be observed in younger patients in association with higher-energy mechanisms and acute disc herniations. Clinically these patients often present with minor abrasions or lacerations on the scalp/forehead and a variable degree of neurological impairment. Suspicion of the injury should prompt MR imaging, even with negative radiographs. The management of these patients is controversial, given a paucity of randomized prospective studies evaluating the outcome of operative versus nonoperative treatment. Early series raised concern about neurological deterioration following acute surgical management, although more recent series have reported otherwise,^87^ with a benefit being noted with operative decompression. Guest *et al.* reported that early surgery was safe and cost-effective in comparison to late surgery (as defined by greater than 24 hours).^88^ They reported an improved motor recovery in patients whose injury was due to a fracture or acute disc herniation, but did not see a similar benefit in the setting of cervical spondylosis. More recent reports have demonstrated a poorer prognosis in patients with advanced age, lower initial American spinal injury association (ASIA) motor score and development of spasticity.^89^ In the only natural history study to date, it was shown that an outcome similar to that reported with surgical decompression is possible with conservative management of central cord syndrome in the spondylotic patient.^90^ In a recent interpretation of the current available literature by Harrop *et al.*,^91^ a general guideline to the management of these injuries was proposed: 1. Patients less than 50 years of age with a traumatic injury and instability warrant operative intervention. 2. Patients less than 50 years of age with an acute disc herniation may benefit from an anterior decompression. 3. The benefit of surgical intervention in “classic” central cord syndrome in elderly spondylotic patients is less clear, and treatment remains at the discretion of the consulting surgeon.^91^ It is also reasonable to add that early surgical intervention appears to be safe in all patient populations. It is the authors' practice to treat patients according to these guidelines, with surgical decompression being performed in the elderly spondylotic patient who is medically fit for surgery and has no evidence of early clinical improvement following the injury. The timing of surgery in these patients is determined by their medical status and not their time of injury.

### Vertebral artery injuries

An injury that can easily be overlooked in the initial evaluation of patients with cervical spine trauma is vertebral artery injury. Vertebral artery injuries may include dissections, occlusions, transections or pseudoaneurysms. The clinical presentation of vertebral artery injuries is diverse; it may include quadriplegia not compatible with a known level of cervical injury, brain stem/ cerebellar infarction, dysphagia, diplopia, blurred vision or nystagmus; although the majority of injuries are asymptomatic. A classic clinical picture is that of “Wallenberg's syndrome,” which is characterized by deficits in CNs V, IX, X, XI; Horner's syndrome; ataxia; dysmetria and contralateral pain/ temperature loss. The incidence of vertebral artery injuries is difficult to determine. In early screening studies, a variety of inclusion criteria were utilized; but the majority included one or more of the following injury patterns: facet dislocations, vertebral body subluxations, transverse foramen fractures and upper c-spine fractures. The incidence was found to range from 16% to 100%^92^ in these initial series of high-risk patients. The screening methods which have been utilized are digital subtraction angiography, which is considered the gold standard although it has a 1% overall complication rate; MRI, which has the advantage of being noninvasive and has a sensitivity/ specificity of 75%/ 67%; and CT angiography, which uses a small contrast bolus and has a sensitivity/ specificity of 68%/ 67%.^93^ In one of the largest screening studies to date, Biffl *et al.* undertook a prospective screening study of all blunt trauma patients at a single institution using digital subtraction angiography.^94^ They reported an incidence of 0.53%, with the majority having suffered a cervical spine fracture. A portion of patients in this study were treated with anticoagulation, but the ability to draw conclusions about the efficacy of treatment was limited by the lack of an experimental design, incomplete followup and small numbers of vertebral artery injuries. Given that the majority of these injuries are asymptomatic, that the gold standard screening method is invasive and that the role of anticoagulation has not been demonstrated to have clear clinical benefits, it is imperative that further research be performed before recommendations can be made regarding the treatment of asymptomatic vertebral artery injuries. Currently the recommendations for management of vertebral artery injuries by the American Association of Neurological Surgeons (AANS) area a) anticoagulation with intravenous heparin in patients with evidence of posterior circulation stroke, b) either observation or anticoagulation in patients with evidence of posterior circulation ischemia, c) observation of patients with no evidence of posterior circulation ischemia.^95^ The authors do not routinely screen asymptomatic patients due to the lack of a widely accepted screening method that is noninvasive. In symptomatic patients, it is our practice to follow the AANS guidelines in the management of these injuries in conjunction with neurosurgical consultation.
